# Exploring the salivary microbiome of children stratified by the oral hygiene index

**DOI:** 10.1371/journal.pone.0185274

**Published:** 2017-09-21

**Authors:** Izumi Mashima, Citra F. Theodorea, Boonyanit Thaweboon, Sroisiri Thaweboon, Frank A. Scannapieco, Futoshi Nakazawa

**Affiliations:** 1 Japan Society for the Promotion of Science, Chiyoda-ku, Tokyo, Japan; 2 Department of Oral Biology, School of Dental Medicine, University at Buffalo, The State University of New York, Buffalo, New York, United States of America; 3 Department of Microbiology, School of Dentistry, Health Sciences University of Hokkaido, Ishikari-Tobetsu, Hokkaido, Japan; 4 Department of Oral Biology, Faculty of Dentistry, Universitas Indonesia, Jakarta, Indonesia; 5 Department of Oral Microbiology, Faculty of Dentistry, Mahidol University, Bangkok, Thailand; Medical University of South Carolina, UNITED STATES

## Abstract

Poor oral hygiene often leads to chronic diseases such as periodontitis and dental caries resulting in substantial economic costs and diminished quality of life in not only adults but also in children. In this study, the salivary microbiome was characterized in a group of children stratified by the Simplified Oral Hygiene Index (OHI-S). Illumina MiSeq high-throughput sequencing based on the 16S rRNA was utilized to analyze 90 salivary samples (24 Good, 31 Moderate and 35 Poor oral hygiene) from a cohort of Thai children. A total of 38,521 OTUs (Operational Taxonomic Units) with a 97% similarity were characterized in all of the salivary samples. Twenty taxonomic groups (Seventeen genera, two families and one class; *Streptococcus*, *Veillonella*, *Gemellaceae*, *Prevotella*, *Rothia*, *Porphyromonas*, *Granulicatella*, *Actinomyces*, TM-7-3, *Leptotrichia*, *Haemophilus*, *Selenomonas*, *Neisseria*, *Megasphaera*, *Capnocytophaga*, *Oribacterium*, *Abiotrophia*, *Lachnospiraceae*, *Peptostreptococcus*, and *Atopobium*) were found in all subjects and constituted 94.5–96.5% of the microbiome. Of these twenty genera, the proportion of *Streptococcus* decreased while *Veillonella* increased with poor oral hygiene status (*P* < 0.05). Furthermore, an unassigned species of *Veillonella*, *Veillonella dispar* and *Veillonella parvula* tended to be elevated in the Poor oral hygiene group. This is the first study demonstrating an important association between increase of *Veillonella* and poor oral hygiene status in children. However, further studies are required to identify the majority of *Veillonella* at species level in salivary microbiome of the Poor oral hygiene group.

## Introduction

Oral bacteria within biofilms associated with teeth cause two of the most common diseases in humans, dental caries and periodontal diseases. Poor oral hygiene resulting in dental caries and periodontal disease can detrimentally affect children's performance in school and achievement in later life, and they can lead individuals to systemic infections, and even death in vulnerable [1]. Children who experience the ill effects of poor oral hygiene are 12 times more inclined to miss school than the individuals who do not [2].

Microbial biofilms play an important role in host homeostasis, metabolic processes, nutrition and protection against deleterious infections [3, 4]. Indeed, disturbance of these communities and changes on their composition have been associated with the development of a variety of diseases [5–7]. Poor oral hygiene status leads to changes in the structure of resident microbial communities driven by an inter-play between the microorganisms and the host response [8]. Hence, an understanding of the composition and ecological events that drive changes in biofilm composition, from good to poor oral hygiene status, is required to develop novel preventive strategies to promote oral health in children.

Recently, high-throughput methods have been developed and employed for epidemiologic investigations relating microbiome profiles to disease risk [9, 10]. As described previously, major approaches to cost-efficient high-throughput characterization of the human microbiome exploit the high variability in microbial 16S ribosomal RNA (rRNA) gene sequence, uniquely found in prokaryotes and considered as a barcode that can be used to identify groups of microbes, including both previously cultivable and so far not-cultivated organisms [11]. The development of now affordable methods has allowed conduct of cohort studies of the human microbiome, providing insight into the diversity and community structure comparing health with disease [11]. These advances have already documented the variability in the oral microbiome as related to dental caries and periodontal diseases [12–14].

In this study, we analyzed the salivary microbiome in Thai children stratified by oral hygiene status. This cohort of children is notable for having only sporadic access to dental care minimal to oral hygiene practice. The bacterial community structure was assessed using high-throughput gene sequences based on bacterial 16S rRNA. The goal was to determine differences in the bacterial profile of saliva of children with poor to good oral hygiene status.

## Materials and methods

### Ethics statement

This study received approval from the Ethics Committee, Mahidol University, Bangkok, Thailand under process number MU-DT/PY-IRB 2015/DT028, and samples were collected at the Dental Hospital, Mahidol University. The participants and their parents were made aware of the objectives and procedures of the study and agreed to participate by providing written informed consent.

### Subjects and sample collection

A total of 90 Thai children were enrolled in the study: 41 males and 49 females (age range: 7 to 15 years). The participants were recruited on March 23^rd^, 2015. All participants were randomly selected by teachers from 191 Border Patrol Police Schools in Thailand after announcement of the project called "Youth Tooth Ambassador" (Youth Tooth Ambassador Project). All participants received an oral examination. They also attended an oral health care course for them to gain knowledge of oral health care as well as general health. The knowledge and information that they received from participating in this activity enabled them to educate their families and communities about the value of oral care. Parents reported their child’s medical history. Children with a history of immunosuppression or systemic diseases (e.g. diabetes and HIV), use of medications that reduce saliva flow, or exposure to antimicrobials in the previous three months were excluded from the study. All other subjects voluntarily joined this study. Regarding sample collection, subjects were informed to refrain from eating, drinking or cleaning their teeth 2 h before their examination. The participants were evaluated by the Simplified Oral Hygiene Index (OHI-S) according to the criteria of Greene & Vermillion [15] and divided into three groups. Six calibrated dentists examined the children for OHI-S and for dental caries using WHO criteria. All examiners took a three-hour training session during which they were calibrated by a senior faculty who served as a gold standard. After training, 10 trial patients were screened by all dentists. The agreement among all examiners in dental caries (DMFT; decayed, missing and filled teeth index) and oral hygiene (OHI-S) was found to be 100% (kappa statistics = 1.0).

The first group (Good oral hygiene) was composed of 24 children with OHI-S scores of 0–1.2. The second group (Moderate oral hygiene) was composed of 31 children with OHI-S scores of 1.3–3.0. The third group (Poor oral hygiene) was composed of 35 children with OHI-S scores of 3.1–6.0. Also at the same time, dental caries experience was measured by using DMFT (for permanent teeth) /dmft (for primary teeth).

After the dental examination, stimulated saliva samples were collected at according to a standardized protocol [16]: each subject was instructed to chew 1 gram of paraffin gum for 1 minute until a solid bolus was formed. Finally, the participant was instructed to expectorate repeatedly for 4 minutes, and for the last 3 minutes saliva was collected in a sterile plastic cup, transferred by a sterile pipette into a sterile plastic tube, which was then stored at—80°C until analysis.

### DNA extraction, library construction and sequencing

Bacterial DNA in saliva was extracted using the Saliva DNA Isolation Kit (NORGEN BIOTEK CORPORATION, Ontario, Canada) according to the manufacture’s instruction. Amplicon libraries were prepared in triplicate by using 16S Metagenomic Sequencing Library Preparation (Preparing 16S Ribosomal RNA Gene Amplicons for the Illumina MiSeq System, illumina^®^) according to the manufacture’s instruction. Briefly, the protocol included the primer pair sequences, 16S amplicon PCR forward primer overhang adapter: 5’-TCGTCGGCAGCGTCAGATGTGTATAAGAGACAGCCTACGGGNGGCWGCAG-3’ and 16S amplicon PCR reverse primer overhang adapter: 5’-GTCTCGTGGGCTCGGAGATGTGTATAAGAGACAGGACTACHVGGGTATCTAATCC-3’, for the V3 and V4 region that created a single amplicon of approximately ~460 bp [17]. After the steps to amplify the V3 and V4 region, the amplicons were gel-purified using DNA Gel Extraction Kit (QIAGEN, Valencia, CA) according to the manufacturer’s instructions. These amplicons were applied to a limited cycle PCR adding Illumina sequencing adapters and dual-index barcodes to the amplicon target using the full complement of Nextera XT indices. After purification of the 16S V3 and V4 amplicon away from free primers and primer dimer species using AMPure XP beads (BECKMAN COULTER, Life Sciences, IN, USA) according to the manufacturer’s instruction, a quality and concentration of these amplicons were controlled by Qubit^®^ 3.0 Fluorometer (Thermo Fisher SCIENTIFIC, MA, USA) and Agilent 2200 TapeStation (Agilent Technologies, CA, USA). They were sequenced on Illumina Miseq platform using paired 300 bp reads and Miseq v3 regents at Hokkaido System Science Co., Ltd. (Sapporo, JAPAN). The ends of each read are overlapped to generate high-quality, full-length reads of the V3 and V4 region.

### Data analysis and taxonomy assignments

All raw sequences were trimmed by using Cutadapt (version 1.1, http://code.google.com/p/cutadapt/) against Illumina adaptor sequences and Trimmomatic (version 0.32, http://www.usadellab.org/cms/?page=trimmomatic) against an average quality score <20. After that, read1/read2 (Pair-ends) was concatenated by using fastq-join (ea-utils version 1.1.2–537, http://code.google.com/p/ea-utils/wiki/FastqJoin). Similar sequences were clustered into operational taxonomic units (OTUs) using UCLUST [18] in QIIME (version 1.8.0, http://qiime.org/) [19] with a 97% similarity cutoff. The most abundant sequence in each OTU was chosen to represent OTU. Chimeras were removed from the representative set after being identified using ChimeraSlayer [20]. The representative sequences were annotated using UCLUST algorism with Greengenes database (version 13_8: 16S rDNA database, http://qiime.org/home_static/dataFiles.html) [21] in QIIME using a minimum identity of 90%.

### Statistical analysis

Sampling effectiveness was evaluated by reflection curve that were drown from a raw OTU table. The UniFrac metric [22] was used to determine the dissimilarity between any pair of bacterial communities. The similarity relationship, assessed using the UniFrac metric, was presented in a PCoA (Principal Coordinate Analysis) plots, drawn using beta_diversity_through_plots.py in QIIME after the data were normalized to an equal number of reads per sample. EMPEROR (http://boocore.github.io/emperor/) was used to visualize 3D PCoA plots. To identify differences of microbial communities between the three groups, analysis of similarities (ANOSIM) [23] and multi-response permutation procedure (MRPP) [24, 25] were performed based on the Bray-Curtis dissimilarity distance matrices using R vegan package (version 2.4–1). In addition, UniFrac metric cluster tree was also constructed to evaluate the differences of microbial communities among subjects using QIIME and visualized by FigTree (version 1.4.3) (http://tree.bio.ed.ac.uk/software/figtree/). Number of OTUs and the Shannon index (alpha diversity) were calculated using alpha_diversity.py also in QIIME after the data were normalized to an equal number of reads per sample according to analysis using beta_diversity_through_plots.py. The Kruskal-Wallis H-test post hoc Mann-Whitney U-test with Bonferroni correction was performed to compare age, OHI-S score, DMFT, dmft, α-diversity and the relative abundances of bacterial genera using ystat2008 software. Fisher’s exact test was conducted to look for differences by sex using R version 3. 3. 2. *P* < 0.05 and *P* < 0.002 were considered statistically significant. Correlational analysis between OHI-S score and DMFT/dmft were evaluated using analysis tool in Excel 2010. Heat-map were created using JAVA Treeview [26] after OTUs were clustered using Cluster 3.0 (http://bonsai.hgc.jp/~mdehoon/software/cluster/software.htm).

### Accession numbers

The obtained sequence data were deposited in DDBJ Sequence Read Archive under accession number DRA005424.

## Results

The demographic and oral data of the study population is shown in Table 1. OHI-S scores of the Poor and the Moderate oral hygiene groups were significantly higher than those of Good oral hygiene group (*P* < 0.002), and also the Poor oral hygiene group had significantly higher OHI-S scores than those of the Moderate oral hygiene group (*P* < 0.002). In addition, DMFT and dmft of the Poor and the Moderate oral hygiene groups were significantly higher compared to the Good oral hygiene groups.

**Table 1:** General and oral condition of the 90 subjects.

	Good oral hygiene (n = 24)	Moderate oral hygiene (n = 31)	Poor oral hygiene (n = 35)
Age (yr)^a^	10.75±1.70	11.28±1.39	10.86±1.48
Sex (Female (%))^b^	14 (58.3)	20 (64.5)	15 (42.9)
OHI-S^a^	0.71±0.43	2.40±0.48^c^	3.53±0.37^c^^,^ ^d^
DMFT^a^	0.75±1.07	1.91±1.46^e^	1.84±1.36^e^
dmft^a^	0.08±0.28	0.95±1.23^f^	1.46±1.36^f^

± shows standard deviations.

^**a**^ Significant differences among groups were evaluated by the Kruskal-Wallis H-test post hoc Mann-Whitney U-test with Bonferroni correction.

^**b**^ Significant differences were evaluated by Fisher’s exact test.

^**c**^ Significantly higher than the Good oral Hygiene group (*P* <0.002)

^**d**^ Significantly higher than the Moderate oral hygiene group

^**e**^ Significantly higher than the Good oral hygiene group (*P* < 0.002)

^**f**^ Significantly higher than the good oral hygiene group (*P* < 0.05 and *P* < 0.002, respectively).

Furthermore, correlational analysis between OHI-S score and DMFT or dmft showed a strong correlation (r^2^ = 0.898 and r^2^ = 0.999, respectively). This result suggested that OHI-S status was strongly associated with caries history.

The variable regions (V3–V4) of the bacterial 16S rRNA gene from DNA extracted from each saliva sample were PCR amplified using barcoded universal primers. Sequencing using the Illumina Miseq instrument produced a dataset comprised of 4,019,174 reads (containing the V3–V4 region), of which 3,521,550 passed quality control tests with an average length of 423 ± 1 bp (S1 Table). These sequences were assigned to 38,521 OTUs with at least 97% similarity.

**Fig 1:**
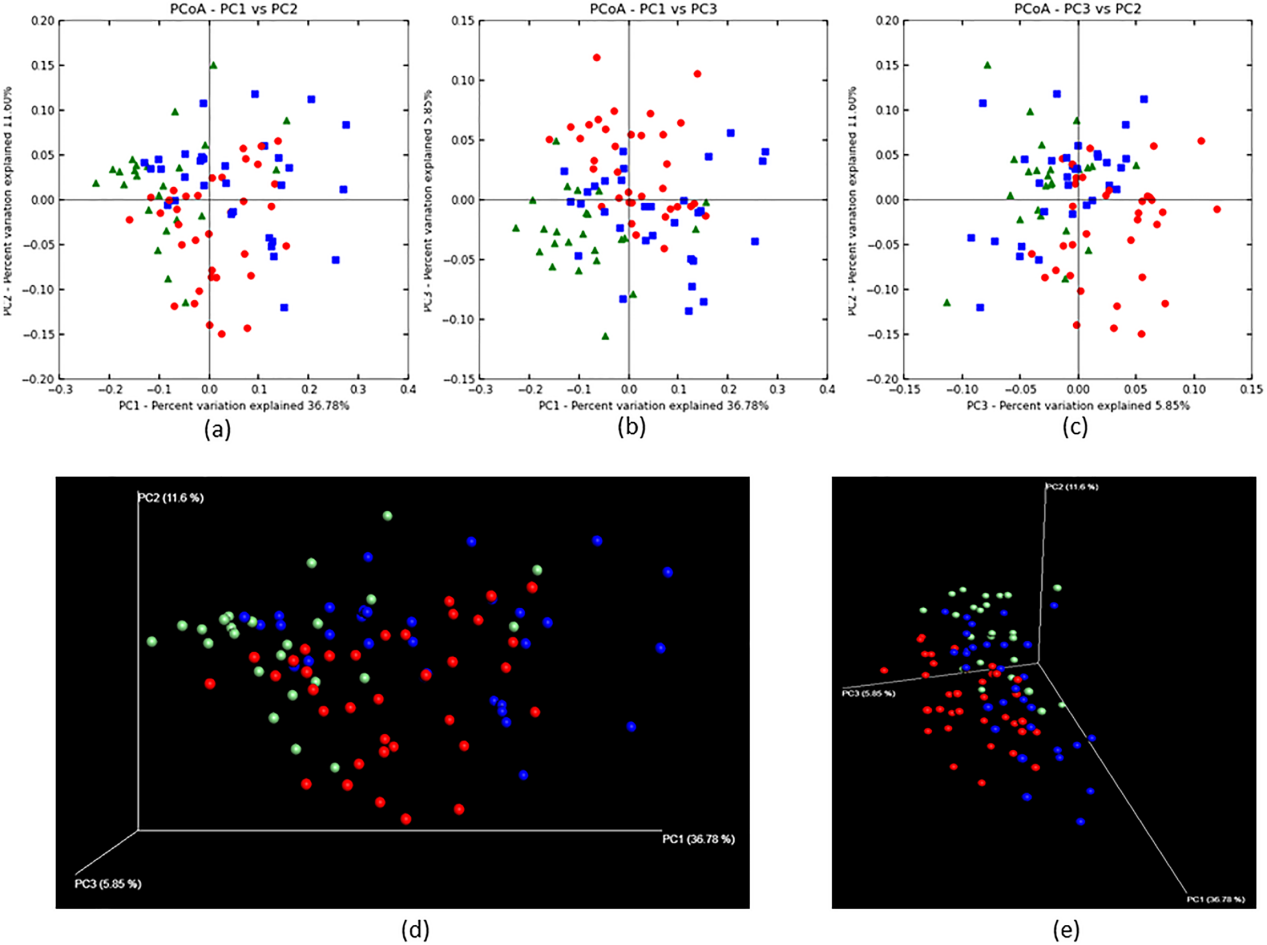
Principal coordinate analysis (PCoA) plot showing the similarity relationships among bacterial community samples from 90 Thai children divided to the three oral hygiene groups using weighted UniFrac distance metric. The green (triangles), blue (squares), and red (circles) indicate the Good, Moderate, and Poor oral hygiene groups, respectively. (a) The two components explained 36.78 and 11.60% of the variance, respectively. (b) The two components explained 36.78 and 5.85% of the variance, respectively. (c) Two components explained 5.85 and 11.60% of the variance, respectively. (d), (e) 3D PCoA plots were visualized by the EMPEROR.

**Fig 2:**
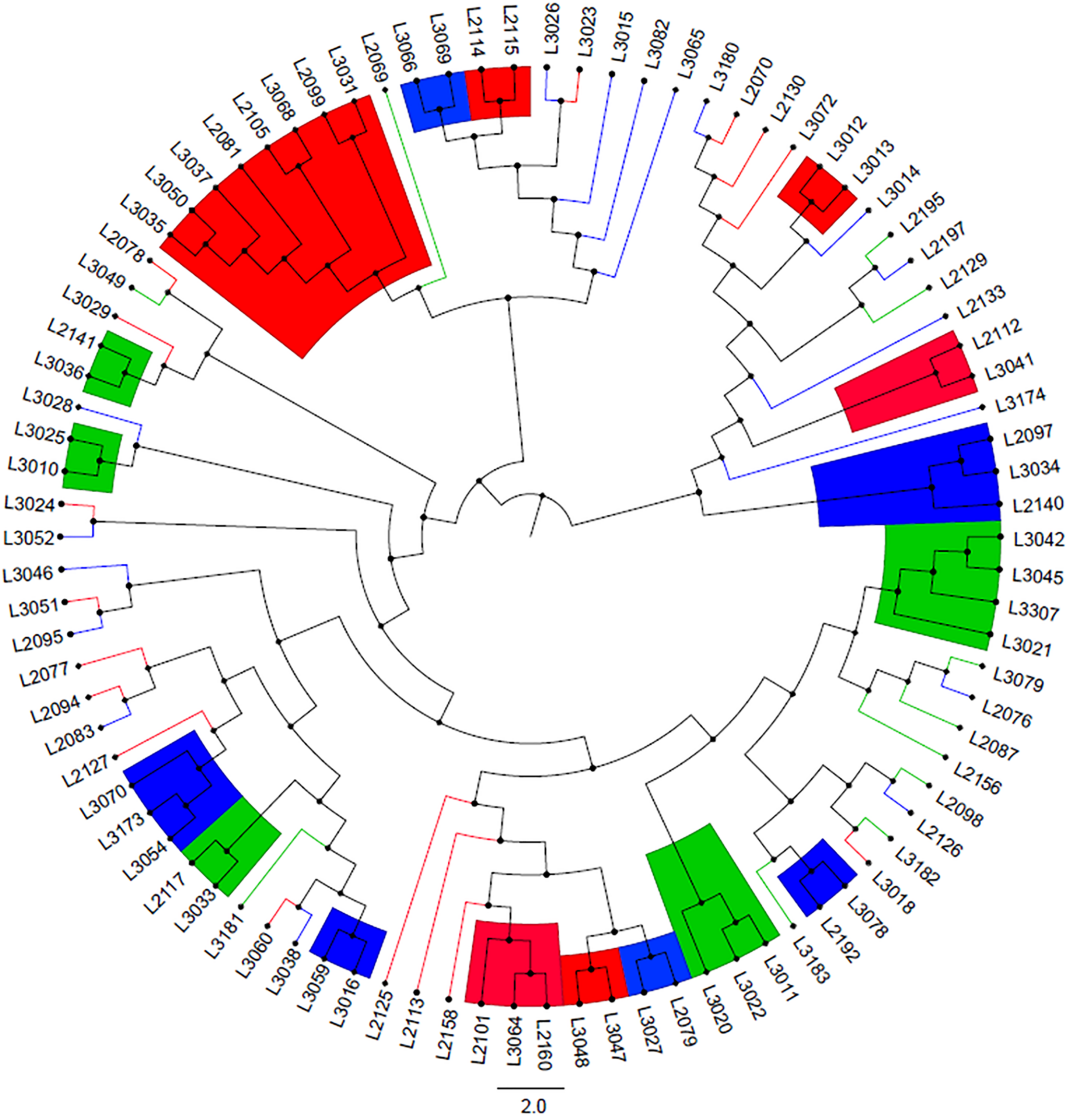
Weighted UniFrac clustering tree of the 90 Thai children individuals. This clustering tree shows the result of weighted UniFrac PCoA plots and visualized the phylogenetic relationship among each salivary microbiome. Each branch represents an individual salivary sample. Samples having similar salivary microbiome compositions are more clustered as highlighted. Green represents Good, blue Moderate and red Poor hygiene branches. Bar length is proportional to phylogenetic distance.

The PCoA plot based on the phylogenetic community analysis with the UniFrac metric were demonstrated overall bacterial community composition in each of the three groups (Fig 1). The PCoA plot showed a partially similar tendency in the distances based on the weighted (counting OTUs and the number of the reads in OTUs of each samples) versions of this metric (Fig 1). Furthermore, the unweighted (counting OTUs of each samples) versions of this metric showed more similar tendency than the weighted version (S1 Fig). However, the differences were confirmed statistically only in weighted versions of this metric using ANOSIM and MRPP analysis (*P* < 0.05, R = 0.75). The data indicated that the microbiome of the three groups of subjects had different community structures. In detail, the PCoA diagram showed that the Good oral hygiene group individuals were localized in the negative direction of principal component 1 (PC1) and in the positive direction of principal component 2 (PC2) relative to the other two groups. On the other hand, the Poor oral hygiene group individuals were localized in the positive direction in principal component 3 (PC3) relative to other two groups. These results suggest that the microbiome in the Good and Poor oral hygiene groups differed relative to the Moderate oral hygiene group. From another point of view, the weighted UniFrac cluster tree showed the microbiome of subjects with poor oral hygiene tended to become similar to each other than to the microbiome of subjects of the other groups (Fig 2). However, these clusters or sub-branches in each group had large phylogenetic distances each other. These findings indicated that salivary microbiome in children differed by OHI-S score, but there were some similar microbiome compositions in each group.

**Fig 3:**
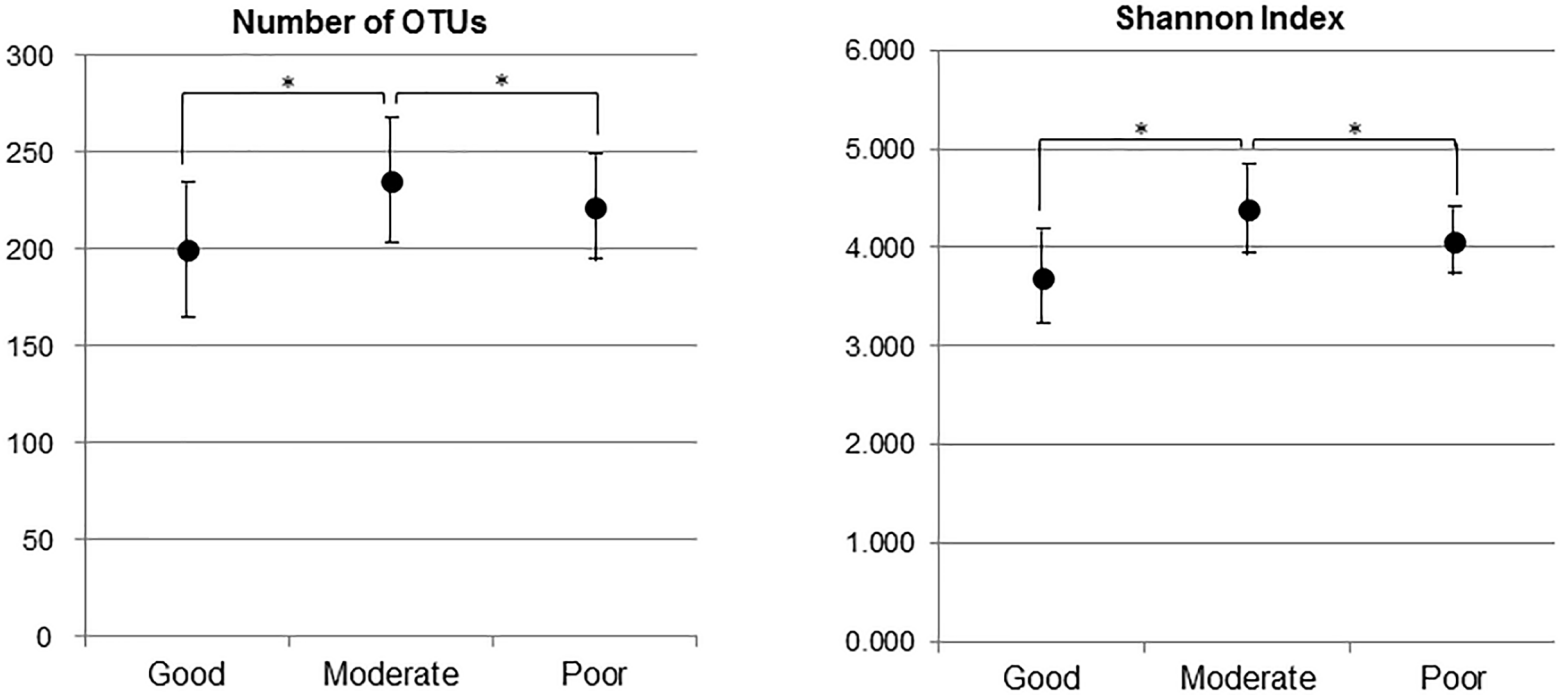
Mean number of operational taxonomic units (OTUs) and Shannon Index in the salivary microbiome of the 90 Thai children divided to the three groups. The significant differences were evaluated by the Kruskal-Wallis H-test post hoc Mann-Whitney U-test with Bonferroni correction (*P* < 0.05). The error bar indicated 95% confidence intervals.

**Fig 4:**
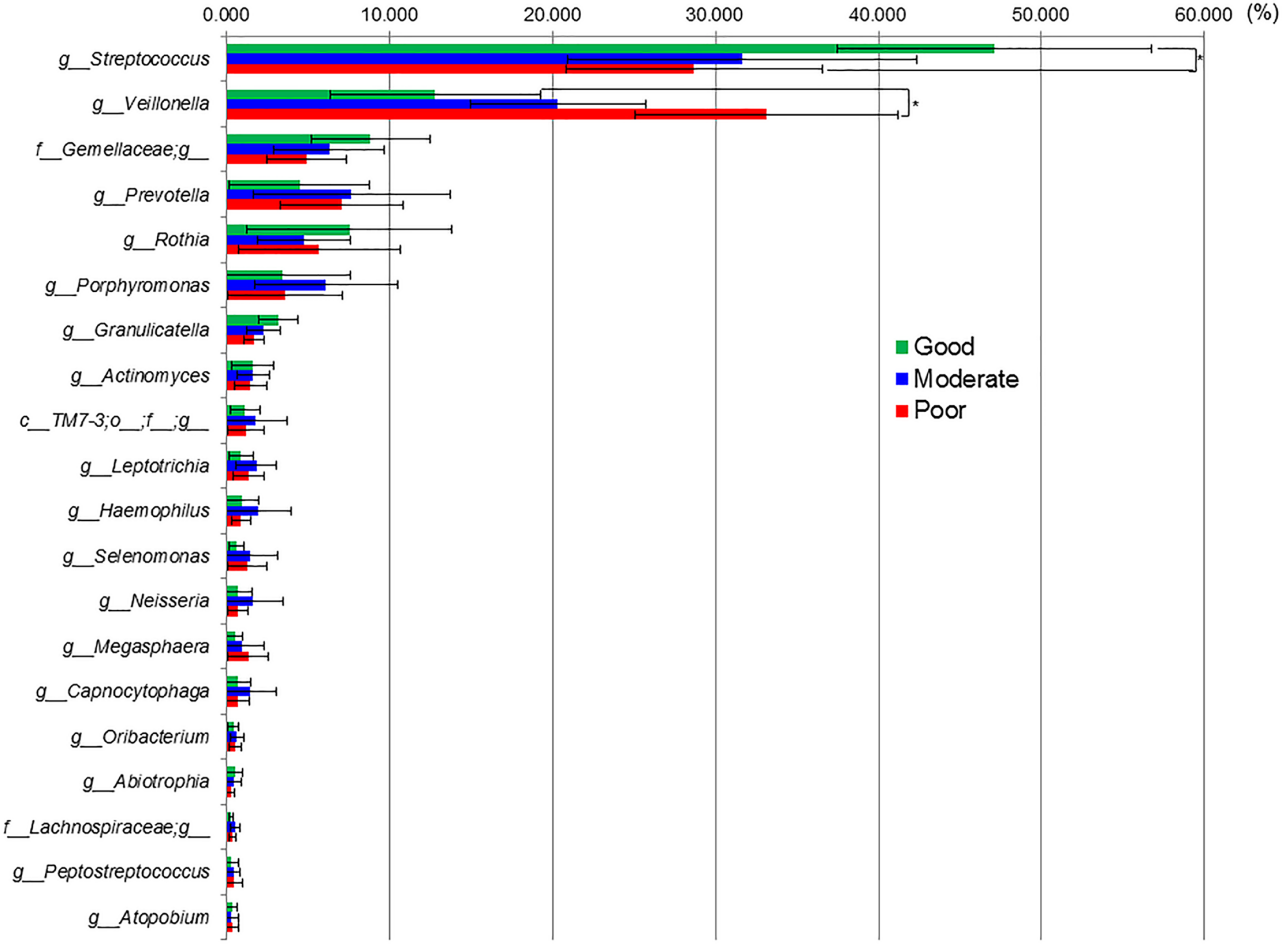
Relative abundances of 20 bacterial genera including upper-taxa among three oral hygiene groups. *C*, *f*, and *g* indicated the class, family and genus, respectively. Some OTUs were not assignable to a genus since the sequence was absent from the database. The significant differences were evaluated using the Kruskal-Wallis H-test post hoc Mann-Whitney U-test with Bonferroni correction (*P* < 0.05). The error bars indicated 95% confidence intervals.

Two indices of alpha diversity indicated that salivary bacterial communities in the Good and Poor oral hygiene group were less diverse compared to the Moderate oral hygiene group (*P* < 0.05) (Fig 3). The dominant phyla (*Firmicutes*, *Bacteroidetes*, and *Actinobacteria*) accounted for 89.74–94.58% of all sequences, *Proteobacteria*, *Fusobacteria* and TM7 for > 1.56% of the sequences in all three groups. The sequences obtained in this study represented 115 genera and 88 upper-level taxa; 64 of the 115 genera and 34 of the 88 upper-level taxa and 225 species were present in the three groups of populations. Twenty genera including three upper level taxa (*Streptococcus*, *Veillonella*, *Gemellaceae*, *Prevotella*, *Rothia*, *Porphyromonas*, *Granulicatella*, *Actinomyces*, TM-7-3, *Leptotrichia*, *Haemophilus*, *Selenomonas*, *Neisseria*, *Megasphaera*, *Capnocytophaga*, *Oribacterium*, *Abiotrophia*, *Lachnospiraceae*, *Peptostreptococcus*, and *Atopobium*) were found in all 90 subjects and constituted 94.5–96.5% of the microbiome. Of these twenty genera, genus *Streptococcus* and genus *Veillonella* showed significant differences (*P* < 0.05) in their proportions between the Good oral hygiene group and the Poor oral hygiene group (Fig 4). More specifically the proportion of *Streptococcus* decreased and those of *Veillonella* increased with worse oral hygiene status.

**Fig 5:**
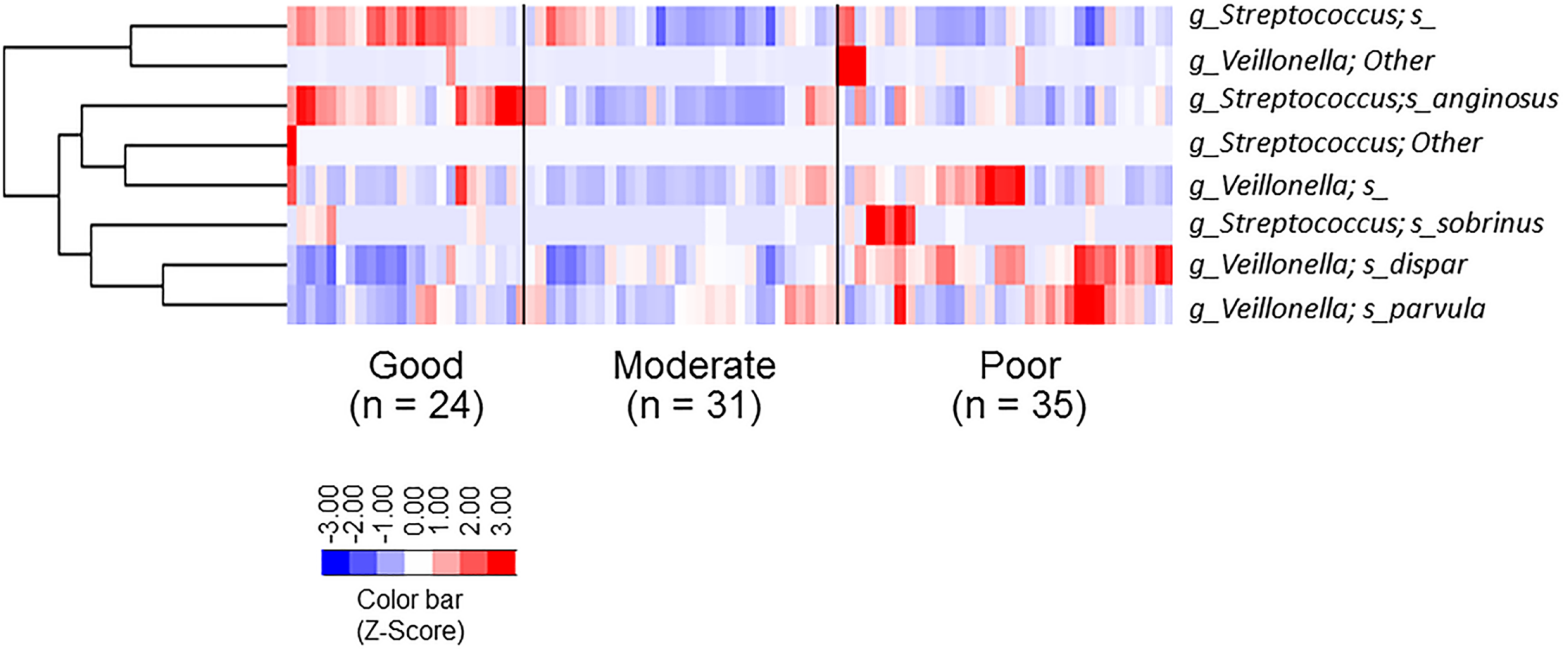
Heatmap displaying species of *Streptococcus* and *Veillonella* distribution patterns in the three oral hygiene groups. To show the distribution of abundant OTUs, the relative abundances of OTU data were normalized to have a mean of 0 and standard deviation of 1 (z-score normalization) at the species level using cluster 3.0. The distribution of the OTUs represented as the color intensity of each grid (blue, low abundance; red, high abundance). All assigned species of genus *Streptococcus* and *Veillonella* in this study were ordered across the three oral hygiene groups. *S* indicates the species. “Other” indicates that no more than half of the sequences in each OTU were obtained from the taxonomic information of that genus. Some OTUs were not assignable to a species since the sequence was absent from the database.

Regarding *Streptococcus* and *Veillonella*, a heat map was constructed to show the relative abundances of OTUs at the species level, including upper-level taxa in all 90 subjects (Fig 5). *Streptococcus; Other*, *Veillonella; Other* and *Streptococcus sobrinus* were associated with all groups (Fig 5). In contrast, *Streptococcus; s_* and *Streptococcus anginosus* (*S*. *anginosus*) were associated strongly with the Good oral hygiene group, and *Veillonella; s_*, *Veillonella dispar* (*V*. *dispar*) and *Veillonella parvula* (*V*. *parvula*) were associated strongly with the Poor oral hygiene group (Fig 5). This finding suggested that *Streptococcus; s_* and *S*. *anginosus* were found in great proportions in the Good oral hygiene groups relative to the Moderate or Poor oral hygiene groups. On the other hand, *Veillonella; s_*, *V*. *dispar* and *V*. *parvula* were found in great proportions in the Poor oral hygiene group relative to the Good or Moderate oral hygiene group.

## Discussion

This study found that the salivary microbiome of children varied depending on oral hygiene status evaluated by OHI-S. The oral cavity consists of a humid environment with relatively constant temperature between 34 and 36°C, neutral pH, and shelters a large variety of microorganisms due to its numerous anatomical structures [27]. Oral bacteria are organized as biofilms that are adapted to every niche within the mouth (for example on the tongue and teeth) as the result of variations in oxygen partial pressure, nutrient availability and so on. The predominant bacteria in saliva are commensal, that detach from the oral tissues [27], for example through bacterial enzymes that degrade biofilm matrix polymers [28]. Also, all epithelial surface cells desquamate, releasing adherent bacteria into the bathing saliva.

The results of the alpha diversity analysis showed that the number of OTUs and Shannon index in the Moderate oral hygiene group were significantly higher than those in other two groups (Fig 3). Pereira et al. reported similar results as the present study, where greater bacterial diversity was found in the saliva of patients with low dental plaque than that of patients with high dental plaque [27]. It is accepted that the number of bacteria in saliva is inversely related to oral hygiene status, however it is not clear if the diversity of bacteria is affected by oral hygiene status. However, further studies are required to clarify if salivary bacterial diversity is related to oral hygiene status.

The microbiome of the subjects of the three groups were dominated by twenty genera including three upper-level taxa, especially *Streptococcus* and *Veillonella* (Fig 4). The salivary microbiome appears to consist disproportionally of microorganisms from the tongue biofilm. Marger et al. reported that the papillate surface of the tongue harbor a microbiome skewed towards anaerobic bacteria such as *Prevotella* and *Veillonella*, whereas the ventral surface bears a microbiome rich in *Streptococci* and *Gemella* [29]. Consistent with the contribution of tongue biofilm populations, the salivary microbiome has been reported to contain a number of genera with the most prevalent or autochthonous being *Prevoella* and *Streptococcus* [30]. These four genera including upper-taxa were found in our results at the top of four higher proportions (Fig 4). Our results in the present study supported these previous reports also in case of children.

This study demonstrates several limitations. First, only salivary samples were collected. Of course, the oral microbiome also includes supra- and sub-gingival plaque that may be more pertinent to dental caries and periodontal inflammation, respectively [31, 32]. Also, correlational analysis between OHI-S score and DMFT or dmft showed a strong correlation. The analysis of supragingival plaque would help to better understand the relationship between oral hygiene status and caries history. In addition, the salivary microbiome as related to diet, socioeconomic status of their parents, geographical location (outside Thailand) and population was not considered in this study. Several studies have reported that dietary treatments were effective to influence the gut microbiome [33, 34]. The salivary microbiome might be influenced by diet as well. The bacterial profile of saliva might also be influenced by socioeconomic status and the geographical location and population [16, 35, 36]. Accordingly, these factors should be considered in future studies.

It has been suggested that many protocols for bacterial DNA isolation from clinical samples is problematic due to non-uniform lysis of bacterial cells, particularly gram positive bacteria [37]. However, Vesty *et al*. [38] recently reported that representation of bacterial genera in plaque and saliva samples did not significantly differ across DNA extraction methods [38]. Furthermore, while Nisiwaki *et al*. [39] reported that the enzymatic lysis method is the preferred method to extract bacterial DNA in stool samples, several commercial DNA extraction kits appear adequate [39]. We choose the Saliva DNA Isolation Kit (NORGEN BIOTEK CORPORATION), which has been used for many studies [40, 41].

Another limitation centers on the use of different hypervariable regions (V1-V9) of 16S rRNA gene for accurate and reliable analysis of salivary microbiome structure, composition and function. Many oral microbiome studies have used the 16S V3-V4 regions because they have been predicted to provide adequate phylogenetic assignment [42–44]. Thus, we choose the V3-V4 regions for analysis of the salivary microbiome. Additionally, the Greengenes database (version 13_8: 16S rDNA database, http://qiime.org/home_static/dataFiles.html) [21] was used to annotate the representative sequences in this study. Human Oral Microbiome Database (HOMD, http://www.homd.org/) [45] has also been commonly used to annotate the sequences in many studies of oral microbiome.

*Streptococcus* species catabolize carbohydrates to short-chain organic acids, such as lactic acid and pyruvic acid [46]. *Veillonella* cannot catabolize sugars; instead, they rely on the fermentation of organic acids to propionic and acetic acids, carbon dioxide, and hydrogen [47]. Thus, a rudimentary food web is established whereby oral *Veillonella* depend upon organic acids produced by oral *Streptococcus* [48, 49]. The proximity of the producer to the consumer could be important in such metabolite transfers [48, 49]. Furthermore, *Streptococcus* participate in biofilm formation as initial colonizer in oral cavity [50] and produce organic acids. After that, the accumulation and maturation of oral biofilm provide anaerobic environment and promote a growth of *Veillonella*, obligate anaerobe, as early colonizer which has central roles to form oral biofilm [50]. It was also reported that the growth of anaerobic organisms such as Veillonellae and Fusobacteria was dependent upon prior growth of aerobic and facultative organisms with a resultant increase in plaque thickness yielding conditions suitable for anaerobic growth [51]. In this study, it was shown that the growth of *Streptococcus*, a facultative anaerobe, may be inhibited by anaerobic conditions in favor of the growth of *Veillonella*. Thus, *Veillonella* is associated with poor oral hygiene status. These shifts in bacterial composition in oral biofilm are reflected in the salivary microbiome. From these results, it was indicated that the proportion of *Veillonella* in salivary microbiome might be a biological indicator for poor oral hygiene and caries activity in children. In several reports regarding salivary and plaque microbiome, *Veillonella* was found in higher proportion in caries or periodontal subjects [14, 35, 52]. According to these results, it was suggested that *Veillonella* tended to be found frequently in microbiome of subjects having poor oral health from children to adults.

Previous studies have reported results of high-throughput microbial gene sequencing based on 16S rRNA to analyze the differences in oral microbiome composition as related to dental caries and periodontal diseases [12–14]. Rajendhran & Gunasekaran reported that some 16S rRNA sequences could be used to assign an unknown organism to a group (genus or family) [53]. *Veillonella* species cannot be differentiated on the basis of 16S rRNA gene sequencing because they share at least 99% of their *rrs* gene nucleotide bases [54, 55]. We previously used a one-step PCR method based on *rpo B* gene sequence to specify oral *Veillonella* species [56]. The present study suggested that *Veillonella*; *Other* was a frequent species and *Veillonella*; *s_*, *V*. *dispar* and *V*. *parvula* were found in greater proportions in saliva from these children with the poor oral hygiene status (Fig 5). Future studies that utilize techniques that can identify *Veillonella* to the species level will validate this finding.

In summary, the present study suggests that differences in salivary bacterial profile may depend on oral hygiene status. However, this could also be influenced by diet, socioeconomic status, as well as the geographic location or population studied [16, 33–36]. This is the first study demonstrating a potentially important association between *Veillonella* and poor oral hygiene status in children, suggesting that *Veillonella* may serve as a biological indicator for poor oral hygiene status.

## Supporting information

S1 Fig **(a)–(e) Principal coordinate analysis (PCoA) plot showing the similarity relationships among bacterial community samples from the 90 Thai children divided to the three oral hygiene groups using unweighted UniFrac distance metric.** The green (triangles), blue (squares), and red (circles) colors showed the Good, Moderate, and Poor oral hygiene groups, respectively. Multiple colors showed the 90 Thai children individuals in (e). (a) The two components explained 6.95 and 6.48% of the variance, respectively. (b) The two components explained 6.95 and 4.01% of the variance, respectively. (c) Two components explained 4.01 and 6.48% of the variance, respectively. (d), (e) 3D PCoA plots were visualized by the EMPEROR.
(TIF)

S1 Table Summary of sequencing analysis. ± shows standard deviations.
(PDF)
