# Supplementary figures and images for: Exploring the salivary microbiome of children stratified by the oral hygiene index
Source: PLoS One. 2017 Sep 21;12(9):e0185274. doi: 10.1371/journal.pone.0185274 (PMC5608389; doi:10.1371/journal.pone.0185274)

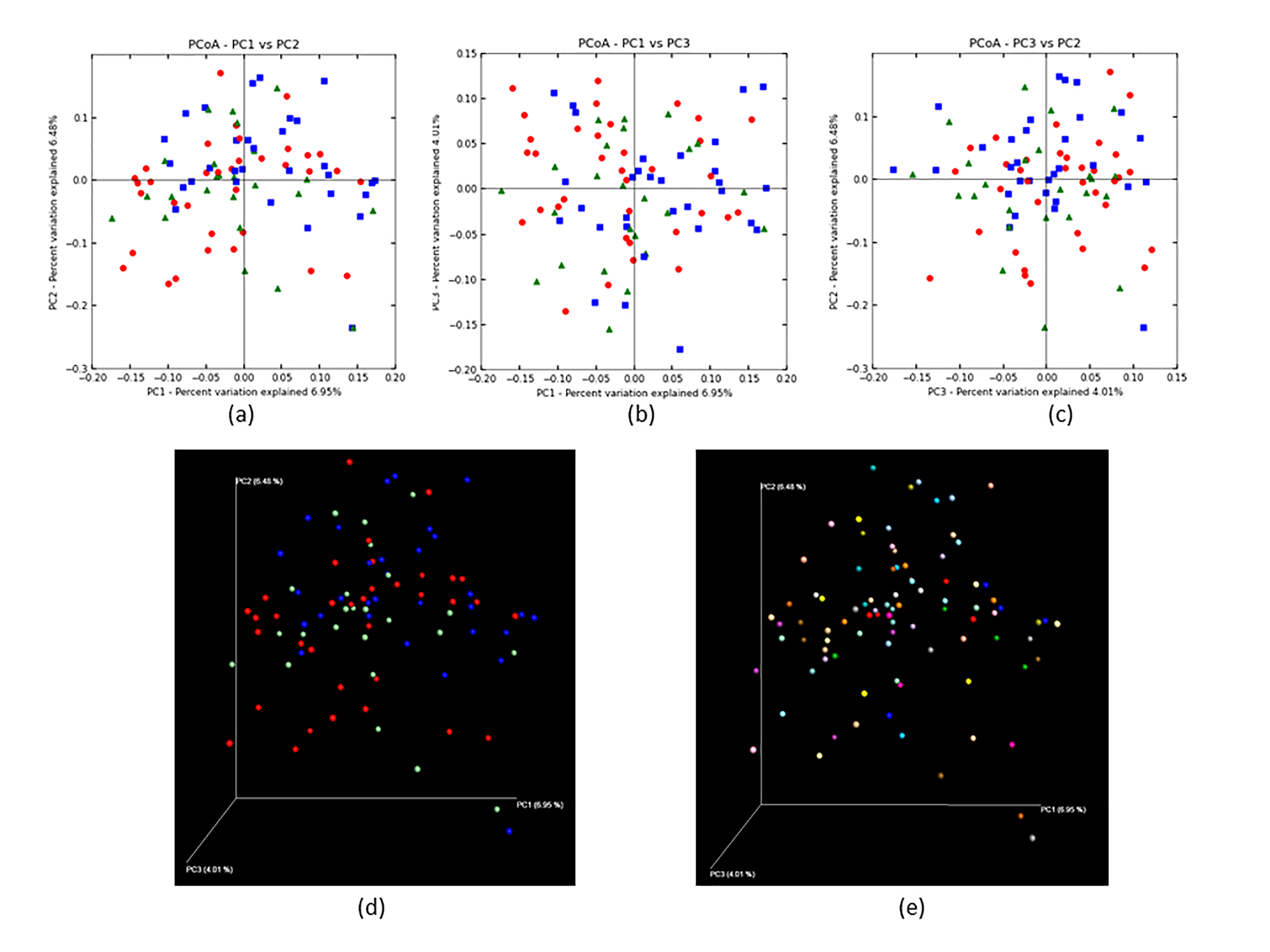

Supplement: S1 Fig — (a)–(e) Principal coordinate analysis (PCoA) plot showing the similarity relationships among bacterial community samples from the 90 Thai children divided to the three oral hygiene groups using unweighted UniFrac distance metric. The green (triangles), blue (squares), and red (circles) colors showed the Good, Moderate, and Poor oral hygiene groups, respectively. Multiple colors showed the 90 Thai children individuals in (e). (a) The two components explained 6.95 and 6.48% of the variance, respectively. (b) The two components explained 6.95 and 4.01% of the variance, respectively. (c) Two components explained 4.01 and 6.48% of the variance, respectively. (d), (e) 3D PCoA plots were visualized by the EMPEROR. (TIF) [file pone.0185274.s001.tif]
