# Supplementary material for: Exploring the salivary microbiome of children stratified by the oral hygiene index
Source: PLoS One. 2017 Sep 21;12(9):e0185274. doi: 10.1371/journal.pone.0185274 (PMC5608389; doi:10.1371/journal.pone.0185274)
Supplement: S1 Table — ± shows standard deviations. (PDF) [file pone.0185274.s002.pdf]

Good oral hygeine (n = 24)			Moderate oral hygeine (n = 31)			Poor oral hygeine (n = 35)		
Sample name	Sample ID	No. of reads	Sample name	Sample ID	No. of reads	Sample name	Sample ID	No. of reads
G-1	L3010	45,522	M-1	L3014	47,612	P-1	L3012	44,744
G-2	L3011	45,260	M-2	L3015	47,194	P-2	L3013	44,124
G-3	L2069	44,978	M-3	L3016	46,560	P-3	L2070	47,410
G-4	L3020	44,610	M-4	L2076	43,174	P-4	L3018	45,644
G-5	L3021	43,698	M-5	L2079	46,904	P-5	L2077	47,944
G-6	L3022	47,576	M-6	L2083	44,826	P-6	L2078	47,426
G-7	L2087	46,766	M-7	L3026	45,508	P-7	L2081	45,856
G-8	L3025	46,000	M-8	L2095	46,624	P-8	L3023	47,060
G-9	L2098	45,090	M-9	L3027	44,992	P-9	L3024	46,380
G-10	L3033	45,820	M-10	L2097	45,608	P-10	L2094	47,158
G-11	L2117	44,978	M-11	L3028	44,454	P-11	L2099	44,410
G-12	L2129	44,978	M-12	L3034	45,216	P-12	L2101	28,728
G-13	L3036	44,340	M-13	L2126	35,576	P-13	L2105	45,474
G-14	L3042	45,196	M-14	L2133	47,002	P-14	L3029	47,922
G-15	L2141	46,810	M-15	L3038	47,282	P-15	L3031	46,778
G-16	L3045	47,690	M-16	L2140	47,354	P-16	L2112	45,546
G-17	L3049	45,622	M-17	L3046	47,174	P-17	L2113	45,338
G-18	L2156	46,682	M-18	L3052	43,830	P-18	L2114	44,822
G-19	L3307	44,290	M-19	L3054	47,038	P-19	L2115	44,306
G-20	L3079	29,572	M-20	L3059	44,454	P-20	L3035	44,858
G-21	L2195	42,290	M-21	L3173	45,540	P-21	L2125	43,518
G-22	L3181	45,406	M-22	L3065	45,324	P-22	L2127	46,000
G-23	L3182	44,842	M-23	L3066	44,766	P-23	L2130	44,554
G-24	L3183	44,372	M-24	L3069	47,286	P-24	L3037	47,618
			M-25	L3070	46,660	P-25	L3041	45,756
			M-26	L3174	44,916	P-26	L3047	46,656
			M-27	L3078	32,080	P-27	L3048	46,140
			M-28	L3180	45,924	P-28	L3050	44,956
			M-29	L3082	17,288	P-29	L3051	44,326
			M-30	L2192	43,278	P-30	L2158	46,084
			M-31	L2197	35,544	P-31	L2160	45,050
						P-32	L3060	47,848
						P-33	L3064	45,870
						P-34	L3068	47,804
						P-35	L3072	45,690
Average		44,683 ± 3,438			43,774 ± 6,134			45,423 ± 3,155
